# Root Causes and Mechanisms of Failure of Wind Turbine Blades: Overview

**DOI:** 10.3390/ma15092959

**Published:** 2022-04-19

**Authors:** Leon Mishnaevsky

**Affiliations:** Department of Wind Energy, Risø Campus, Technical University of Denmark, Frederiksborgvej 399, 4000 Roskilde, Denmark; lemi@dtu.dk

**Keywords:** wind energy, wind turbine blades, maintenance, structural health monitoring

## Abstract

A review of the root causes and mechanisms of damage and failure to wind turbine blades is presented in this paper. In particular, the mechanisms of leading edge erosion, adhesive joint degradation, trailing edge failure, buckling and blade collapse phenomena are considered. Methods of investigation of different damage mechanisms are reviewed, including full scale testing, post-mortem analysis, incident reports, computational simulations and sub-component testing. The most endangered regions of blades include the protruding parts (tip, leading edges), tapered and transitional areas and bond lines/adhesives. Computational models of different blade damage mechanisms are discussed. The role of manufacturing defects (voids, debonding, waviness, other deviations) for the failure mechanisms of wind turbine blades is highlighted. It is concluded that the strength and durability of wind turbine blades is controlled to a large degree by the strength of adhesive joints, interfaces and thin layers (interlaminar layers, adhesives) in the blade. Possible solutions to mitigate various blade damage mechanisms are discussed.

## 1. Introduction

Wind energy is one of the fastest growing renewable energy technologies. Over the last decades, the global installed wind energy generation capacity has increased drastically from 7.5 GW in 1997 to 564 GW by 2018. In 2019, 60 GW of new capacity was added. In 2020, the wind sector installed 93 GW. In 2020, the global installed offshore wind capacity reached around 5% of the total wind capacity [1,2]. A large expansion of wind energy is planned for the next decades. The global wind power market is expected to reach 69.7 GW by 2027 [3].

**Figure 1:**
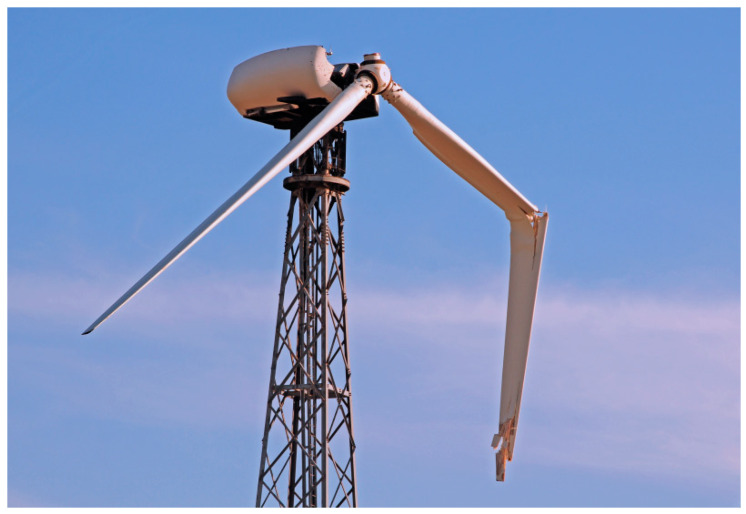
Wind turbine with broken blades. The photo from Bigstock© collection is reproduced according to the Bigstock content usage agreement.

However, the maintenance of wind turbines remains an important cost factor, influencing the energy price and competitiveness of renewable energy [4]. For instance, onshore wind farm operators spent around USD 15 billion on operations and maintenance services in 2019, 57% of which was on unplanned repairs [5]. Further, the number of wind turbines reaching 20 years of operation annually, for instance, in Spain and Germany will be of the order of 800 per year in the next decade [6]. An ageing fleet leads to increased repair requirements [7,8]. With an estimated 700,000 blades in operation globally, there are, on average, 3800 incidents of blade failure each year. The total repair budgets in Europe increased from 2019 to 2020 from USD 4.7 to 8.6 mil, and unplanned repairs also increased slightly in Europe (2%) and even by 10% in America [6]. Failure events of different degrees take place relatively often. Figure 1 shows a wind turbine with broken blades.

Wind turbine blades are subject to complex environmental and mechanical loading during their service time, including cyclic deformation, rain, sand and contaminants causing erosion, icing, high moisture and temperature variations, but also extraordinary events, such as transportation damage, lightning strikes and bird impacts [9,10,11,12]. In order to reduce and optimize the maintenance costs, a detailed understanding of the degradation and failure mechanisms of wind turbines is required. This is important for a reliable prediction of failure events, the planning of maintenance activities and the mitigation of the degradation processes. For wind turbine blades, the lifetime extension is one of best strategies of their use after 25 years of service time [7].

In this paper, the mechanisms of degradation and failure of wind turbine blades under service conditions are reviewed, with a view also on the role of manufacturing defects and possible solutions.

## 2. Wind Turbine Blade Failure Mechanisms

### 2.1. Methods of Analysis of Mechanisms of Wind Turbine Blade Failure

Wind turbine blade damage can be classified as surface damage (microcracks on the surface and coatings), resin and/or interface damage (delamination, defects in resin) and structural element damage (with broken or kinked fibers) [10]. The surface damage can be caused by erosion (rain erosion, sand, hail), or small object impacts. The damaged, rough surface can reduce the aerodynamic performance of blades and energy generation. It does not prevent the wind turbine from functioning, but the surface defects grow and develop and can lead to structural damage of the blade.

Generally, failure mechanisms of wind turbine blades are analyzed using the following main methods:

- Post-mortem analysis of failed blades;
- Full scale testing of blades in laboratories with video-observation and structural health monitoring;
- Analysis of databases and collections of incident reports;
- Direct monitoring of blade deformation and degradation during service (for instance, using non-destructive testing and structural health monitoring methods);
- Testing design of subcomponents (e.g., beam), reproducing parts or elements of the blades (e.g., joints or sandwiches);
- Computational modelling of blade deformation and damage.

**Post-mortem analysis** of failed or damaged blades (either test blades or blades taken from old or damaged wind turbines) is the most obvious approach to explore the blade failure mechanisms. A visual analysis of failed blades, the mechanical testing of used blade parts or their microscopic study allow an analysis of the root causes of failure. Marin et al. [13,14] studied the reasons for the damage to 300 kW wind turbine blades by slicing and inspecting the damaged zone of the blade. They observed cracks in the root to the airfoil transition area and manufacturing defects (lack of resin). The failure occurred due to fatigue mechanisms, starting from a surface crack which appeared around the weakest point (corner between the cover and the root) and developed through the coating into delamination between this layer and the laminate. Later, the surface crack together with the effect of the thickness change triggered the crack in the laminate and full failure.

Specifically with a view to the surface degradation of blades, Fæster, Mishnaevsky and colleagues [15,16] studied samples from blades from the Vindeby wind farm on the Danish island of Lolland, and carried out a microscopic analysis of the defects and damage in the eroded blades. Using the post-mortem microscopic analysis, they could establish the microscale mechanisms of the surface degradation of wind turbine blades (for more details see Section 4).

The post-mortem analysis of decommissioned blades can allow a quick identification of the failure mechanisms for specific blades, location and sites. However, it is often difficult to make more general conclusions and observe tendencies due to the limited knowledge of the blade service history in each specific case.

Another approach is based on an **analysis of a database of incident reports** and failure frequencies analysis. Referring to the WindStats Newsletter, HSE Electrical Incident Database, RenewableUK “lessons learnt” database and US National Renewable Energy Laboratory (NREL) data, Robinson et al. [17] provided the following classification of blade failure mechanisms: root connection failure, catastrophic structural buckling or separation, leading edge, trailing edge, or other bond separation, lightning damage, erosion and failure at the outboard aerodynamic device. The authors of Ref. [17] also classified blade failure into catastrophic (breaking primary structure, failure of structural element, separation of parts), functional (stiffness reduction, permanent deformation, change of shape) and superficial (small cracks, e.g., in the bondline or gel coat, pant flaking, surface bubbles, delaminations, minor buckling).

Branner and Ghadirian [18] reviewed several databases on blade unavailability and downtimes, including German WMEP and LWK, Finnish VTT, Swedish Vindsat, US CREW and others. They analyzed wind turbine blade failure mechanisms and classified them according to the urgency of repair and the reasons for the damage. Apart from force majeure situations when the wind turbines cannot function anymore (lightning, tower hit by blade, transport damage, missing external parts), they listed interlaminar failure, transverse cracks from the trailing edge and on the blade surface, fatigue failure in the root connection as critical damage requiring the turbine to stop. Delamination due to a faulty injection, flaking and cracking of the coating, fatigue failure in the bond lines and in the root transition area, longitudinal cracks in the trailing edge are the damage types that should be repaired at an opportunity or as soon as possible.

Carroll and colleagues [9] presented an analysis of the failure rates of wind turbines, based on ~350 offshore wind turbines located throughout Europe. They also considered blades, estimating the frequency and repair requirements for minor and major blade defects. They estimated the minor repair requirement for blades as 0.456/turbine/year and major repair requirements as 0.01 turbine/year.

**Figure 2:**
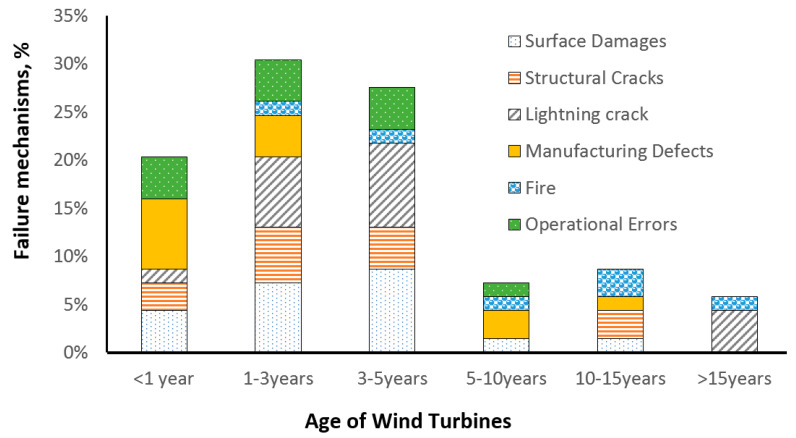
Results of the survey of blade service companies: Frequency of wind turbine blade failure mechanisms depending on the age of wind turbines. Reprinted from [19] with kind permission from John Wiley and Sons.

Boopathi, Sumatraa and colleagues [19] carried out a survey among the wind turbine service teams regarding the frequency of the observed damage mechanisms in different regions in Europe and India. Figure 2 shows the results of the survey of blade service companies, presenting the frequency of wind turbine blade failure mechanisms depending on the age of wind turbines based on data from [19]. They observed that leading edge erosion and lightning strikes are the two most often observed damage mechanisms. Leading edge erosion can occur from the first year after wind turbine installation. The lightning strike is observed especially often in monsoon areas. In Europe, fire and even operational errors determine the failure of wind turbines, reaching 5…10 years and more. In India, the wind energy industry is generally younger and less “crowded” than in Europe, and therefore, operational errors and manufacturing defects are among the main failure mechanisms of recently installed wind turbines, even after installation or 1–3 years old. That leads to structural failure even in young, recently installed wind turbines (1–5 years, which is not observed in Europe).

This approach has the advantage of a bigger data volume (as compared with difficult and labor consuming post-mortem studies). The single observations are typically limited, but this is partially compensated by larger data volumes.

**Full scale testing of blades in a laboratory** should allow loading conditions to be varied, thus establishing relationships between blade failure mechanisms and loading cases. This can open the possibility of optimizing the blade materials and elements, with a view on specific failure mechanisms. Full scale testing of blades is carried out both under dynamic and static loading, following the requirements in the IEC 61400-23 standard on full-scale testing [20]. Sørensen et al. [21,22] carried out a full scale failure testing of a 25 m wind turbine blade under flapwise loading, stopping the loading at every sign of damage and analyzing the damaged sites. They also carried out a post-mortem analysis of the blades. The authors identified the following damage mechanisms of wind turbine blades: damage in the adhesive layer/bond joining skin and the main spar flanges (skin/adhesive debonding or main spar adhesive debonding), also including buckling induced skin/adhesive debonding), damage in the adhesive layer joining up- and downwind skins along the leading and trailing edges, damage in the interface between the face and core in the sandwich panels in the skins and main spar web, internal damage in the laminates in the skin or main spar flanges (tensile or compressive delamination), splitting and fracture of the fibers in the skin laminates or main spar, buckling of the skin due to damage in the bond between the skin and main spar under compression, cracks in the gelcoat and peeling off of the gelcoat from the skin.

Lee and Park [23] tested 48.3 m wind turbine blades and analyzed the residual strength of the fatigue-damaged wind turbine blade. The specimens were loaded sequentially along the positive flapwise, the positive edgewise, and the negative flapwise direction. The main conclusion was that the delamination of fatigue-damaged trailing edge layers was the root cause of the blade failure.

Chen and colleagues [24] studied the mechanisms of collapse of a 30 m blade. They used draw-wire displacement transducers to record the displacement of the blade, and three video cameras to capture the collapse process of the blade. Chen and colleagues concluded that while the torsional load does not have strong effect on blade failure, increasing the blade size reduces the blades’ torsional eigenfrequency. This will result in a coupling torsional mode with lower bending modes, potentially leading to structural failure.

The advantage of full-scale blade testing is that it allows new wind turbines, manufactured from new materials to be tested and also allows the use of newer technologies. However, only very idealized loading conditions can be reproduced in the laboratory full scale tests.

**Direct monitoring of the deformation and damage** in wind turbine blades can be carried out using methods of non-destructive testing and structural health monitoring methods [25]. Sensors are attached or embedded in the blades and the deformation and damage events are monitored. While structural health monitoring is typically developed for blade control, it can be also used to understand the failure mechanisms. Such experiments are undertaken, first of all, with a view on the surface erosion of blades. A more detailed review is provided elsewhere [25].

The sub-component testing is used typically for a better understanding of local effects, see Section 3.

While the **computational modelling of the degradation of wind turbine blades** is one of the most efficient approaches to the analysis of failure mechanisms, the models typically include some pre-defined and assumed damage mechanisms. For instance, Shokrieh and Raffiee [26] simulated fatigue damage in a wind turbine blade, using the so-called accumulated fatigue damage modelling approach. They used a static analysis to obtain the critical zone of the blade, which is located on the upper flange of the spars, and then simulated the fatigue crack growth in the critical zone. Raman et al. [27] carried out a numerical simulation of a wind turbine blade under static bending and torsion load and sought to determine the weak areas/areas of high stress concentration in the blade. They observed that the root section and trailing edge were critical zones in the blades. Different specific blade failure mechanisms, studied in computational models, are discussed in Section 3.

Different methods of investigations allow a better understanding of the various aspects and factors influencing the blade damage mechanisms. The analysis of incident reports leads to a better understanding of the effect of real service conditions, location, weather and manufacturing conditions on the damage mechanisms. However, this analysis has an apparent time gap: the observations made on 10, 15 or even 20 years old wind turbines are not always applicable to wind turbines which are manufactured today. Both materials, quality control, technologies and sizes can be different.

Full scale blade testing in laboratories is quite expensive, applicable only to relatively small wind turbines, does not reflect real service conditions and uses laboratory-manufactured materials and structures, which might have fewer or different manufacturing defects than those exploited in the field.

Computational models are quite efficient and have a wide application range. However, their application requires a preliminary knowledge of the expected damage mechanisms.

Generally, the combination of all or several of the listed approaches can be the best approach to obtain a better understanding of real blade damage mechanisms and the possibilities of their mitigation.

### 2.2. Critical Areas of the Wind Turbine Blade

Several regions of wind turbine blades are especially susceptible to degradation, among them, regions subject to the most intensive loading (tip, leading edge), transitional regions (e.g., area of transition from the cylinder to the aerofoil and plydrop), dissimilar and interface-containing regions (with adhesive layers, e.g., trailing edge). According to Refs. [28,29,30,31,32,33], near the root (30–35% of the chord length from the root) and near the tip (70% in chord length from the blade root), the root of the blade, the maximum chord and the upper spar cap/flange of the spar, but also the trailing edge on the high pressure side and on the leading edge are the regions which are damaged most often.

Let us look at the main mechanisms of damage in these regions:

**Blade tip:** Since the speed at the tip is much greater than at the root, erosion with resulting damage to the leading or trailing edges is much more intensive near the tip. Moreover, lightning can initially strike blades near the tip and then can develop into the separation of high-pressure and low-pressure skins near the tip or the separation of the skins from the shear webs [17].

**Leading edge.** The leading edge of a wind turbine blade is commonly subject to rain droplets, hail, sand and successive impacts. If the surface erosion is not repaired quickly, it can lead to the cracking of the laminates or it will allow water penetration into the bond line [17].

**Trailing edge.** The trailing edge of wind turbine blades can fail by debonding in the adhesive joint (e.g., due to peeling stress [33]) and/or by buckling of sandwich panels. The failure is mainly controlled by edgewise moments and influenced by flapwise and torsion moments [34]. Buckling can lead to the final failure of the trailing edge adhesive joint well below the expected maximum load [35]. Ataya and Ahmed [36] reported the results of the inspection of a number of trailing edges of blades of 100- and 300-KW wind turbines and observed both longitudinal cracks along the trailing edge and transverse cracks.

**Tapered areas, plydrop (thickness transitions), root region:** Other potentially damageable regions are the mid spans in the root region of the blade, in the area of transition from cylinder to an aerofoil, due to the sharp transition from thick laminate to fine geometry and large laminate panels near the maximum chord which can be buckling-sensitive [17]. Local stress concentration in tapered areas, with thickness transitions, can lead to ply delamination at relatively low applied strains under fatigue loading due to interlaminar and transverse shear effects in the matrix [37].

**Adhesive joints/bond lines:** Joints at the leading and trailing edges, between the shell and main spar, between the spar cap and internal stiffeners can become damaged or debond, and this can lead to the buckling of the structures [17,38]. Blade collapse can occur if the shear web is detached from the shell and buckles. According to [29], the most often observed failure mechanism of blades are transverse cracks in the max chord region (initiated as a skin disbond from the sandwich core) and the disbond of the aft shear web from the blade shell in the root-transition zone (triggered by peeling stresses in the adhesive bondlines). These effects are enhanced by manufacturing defects and torsional loads on the blade [30].

**Figure 3:**
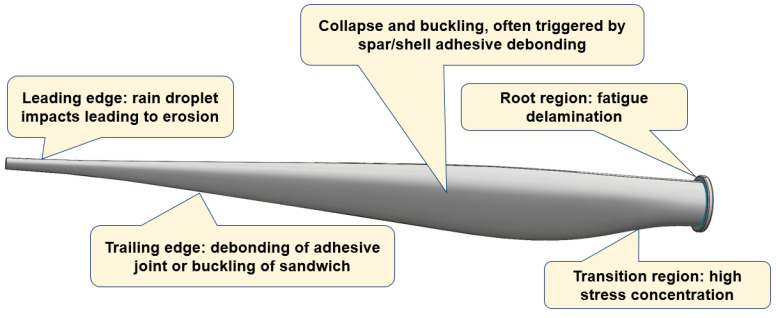
Schema of locations of often-observed damage mechanisms of a wind turbine blade [3,7].

Therefore, the most endangered regions of the blades include the outstanding and high velocity region (tip), the transitional and tapered areas and the interface regions. Figure 3 shows a schema of the locations of the often-observed damage mechanisms of a wind turbine blade.

The general tendency now is to use larger wind turbine blades, often located offshore. Thus, the question of the influence of the size of the blades on their damage mechanism becomes a critical question. Jensen and colleagues [31,32] studied the effect of the large size of blades on the loads and damaged mechanisms. They listed the failure modes which change when blades become longer including, tip deflection, transverse shear distortion and web failure but also (more important with increasing size) fatigue failure in the root connection, in the root transition area and in the bondlines, flutter and trailing edge buckling. According to [31,32], different loads are responsible for different critical areas: aerodynamic loads for the cap, gravity load for the root, flapwise bending for the Brazier effect (cross-section ovalization of cap, bending-induced in-plane warping) and buckling of the cap and tip deflection, edgewise bending for stiffness resonance in the root transition, torsion for the flutter, combination of flapwise bending and edgewise for buckling and the distortion of the cap and cross section, and flapwise bending and edgewise and torsion for distortion and flutter. They also listed scaling laws, with the fourth degree for fatigue in the root connection and root transition area, and the third degree of length for bondlines and trailing edge buckling.

Thus, the most endangered regions of a wind turbine blade are the protruding parts (tip, leading edges), the tapered, transitional areas and the bond lines. Strengthening these regions can drastically increase the durability and lifetime of wind turbine blades.

## 3. Mechanisms of Wind Turbine Blade Failure: Microscale Effects and Root Cause Investigations

In this section, we review the main experimental and computational investigations, seeking to understand the root causes of main failure mechanisms of wind turbine blades.

### 3.1. Leading Edge Erosion

Figure 2 shows the frequency of observing different blade damage mechanisms over years, according to the survey of service companies [19]. It can be seen that surface erosion is the most often observed damage mechanism (for those turbines that survived the risks caused by initial manufacturing defects during the first years and were not subject to lightning strikes). In fact, it is above the structural failure risks, which combine spar buckling, trailing edge failure and adhesive joint failure (i.e., most other failure mechanisms). Surface erosion is also the damage mechanism which most often requires repair and is most expensive for wind turbine owners [4]. According to [4], the costs of minor repairs of wind turbine blades are more than 12 times higher than the costs of major repairs or replacements.

A widely used approach for the erosion testing involves a so-called rain erosion tester, for instance, provided by R & D Test Systems, based on a 3-bladed helicopter principle [39,40]. This test corresponds to DNV GL’s Recommended Practice (DNVGL-RP-0171). Another approach, based on the interrupted jet approach, is the Pulsating Jet Erosion test rig, developed at EADS. Further, the multiple impact jet apparatus (MIJA) and solid projectile impact tester can be used to evaluate the erosion processes in materials. Another technique is the single point impact fatigue testing (SPIFT), in which rubber balls are consecutively shot in the same location of a specimen surface, which is monitored by digital microscope camera [40].

**Figure 4:**
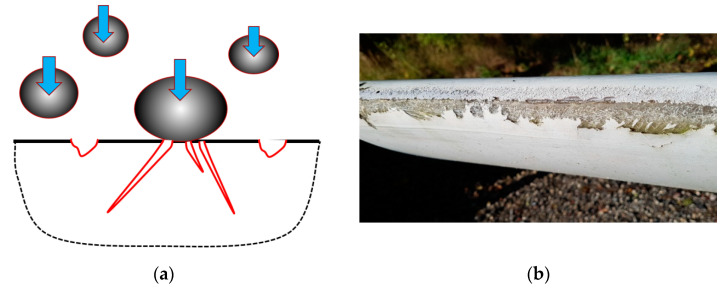
Schema of leading edge erosion of blades (**a**) and eroded blade (**b**).

Figure 4 shows a mechanism of the leading edge erosion of blades, and a micrograph of severely eroded blade. Due to the complexity and microscale and multiphysical mechanisms of erosion, the relationships between laboratory studies and field observations are not straightforward, and computational models are often required to interpret and link the test observations with the field results.

Several models of rain erosion have been developed, from phenomenological formulas (e.g., Springer model [41]) to complex multistep models [42,43]. A computational model of leading edge erosion typically includes the following steps: rain scenario (or for simplification, the modelling of a single droplet), the modelling of the impact contact between the droplet and surface, an analysis of the stress evolution in the coating and laminate, the modelling of the materials’ degradation over time and an evaluation of the effect of the changing surface profile on energy production and the performance of the blade. The rain density, droplet size distribution and velocity are estimated from observations, experimental data or probabilistic [44] or stochastic rain scenarios [42]. For the analytical estimation of the pressure on the coating, the water hammer equation, linking pressure on the surface with the liquid impact velocity and liquid density, is often used in simple models. Several finite element (FE) models have been developed to simulate the raindrop contact and wave propagation in the coatings [43,45,46,47,48,49]. Adler et al. [45] simulated water droplets impacting thick compliant coatings. The development of deep craters was observed in impacted polyurethane coatings which probably altered the evolving water drop shape. Further, it was shown that an impact by a single water droplet cannot initiate failure for a polyurethane coating. Keegan et al. [46] used the combined Eulerian/Lagrangian approach and Explicit Dynamics tool of the FE software ANSYS to simulate the stresses in the composite and the evolution of the droplet shape. Amirzadeh and colleagues [42] carried out an FE transient stress analysis for various raindrop sizes. The entire layup structure and only the gelcoat layer with a rigid bottom were modelled and compared. They estimated fatigue damage as a superposition of damage from individual raindrops, using the Miner–Palmer fatigue rule and the rainflow count. The authors developed a computational framework for the estimation of a lifetime of anti-erosion coatings. Douagou-Rad and Mishnaevsky, Jr. [48,49] used similar methods and developed a fatigue degradation prediction code based on the combined Eulerian–Lagragian technique (CEL) and the Smoothed-particle hydrodynamics (SPH) method, critical plane models (i.e., assuming that fatigue damage accumulates on a specific plane in the material, denoted the “critical plane”) using the Miner–Palmer fatigue rule and the rainflow count. The critical plane model allows the simulation of multiaxial fatigue. As a result, they estimated the damage distribution in the material, and observed that the shape of droplet, the water layer on the coating surface and the roughness of surface have a strong influence on the surface degradation of blades.

Generally, the surface erosion of wind turbine blades (first of all leading edge erosion) is a complex multiphysical process, strongly influenced by random factors (rain conditions, rain properties, defects in the coatings). Still, the erosion (as said) is most often observed and is the earliest observed damage mechanism of wind turbine blades (1…2 years after installation [19]), which can lead to a reduction in the annual energy production of wind turbines (5% and more) and a reduction in further damage in the laminates.

### 3.2. Tapered Areas and Plydrop

**Figure 5:**
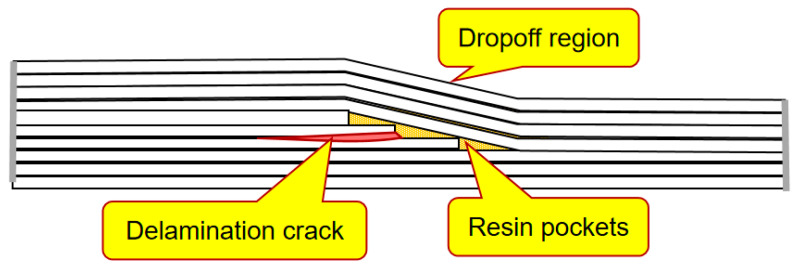
Schema of a delamination crack in the plydrop region.

Tapered areas and plydrops in the blades are necessary in the blades to ensure an optimal, aerodynamic shape. Plydrops are formed by terminating some of composite plies. The tapered structures create local stress concentrations, in particular, interlaminar stresses in the vicinity of the ply drop-off which can lead to the delamination of plies and the failure of these regions [50]. Figure 5 shows a schema of a delamination crack in the plydrop region, starting from a resin pocket.

In a number of studies, the effect of the thickness and the amount of dropped plies on the stress concentration, delamination and fatigue behavior were studied. In their study of beam elements representing the blade substructures, Mandell et al. [51] tested beams with ply-drops in flanges. They observed that dropping more than one ply at the same location increases the delamination tendency.

Cairn and colleagues [52] carried out a systematic testing of fiberglass specimens with plydrops with different geometries (exterior and interior plydrop, thicker/thinner laminates, multiple plydrops). They observed that thicker laminates and internal plydrops lead to a better delamination resistance (as compared to thinner and external ply drops, respectively), while several dropped plies increase the delamination growth rate, as compared with a single plydrop.

Shim [53] studied the mechanisms of delamination and fatigue near ply drop-offs computationally. He also identified two physical mechanisms controlling the interlaminar stresses in laminates with ply drop-offs, namely, the so-called termination effect, caused by the load transfer from the terminated plies to the continuous plies and the offset effect, caused by the redistribution of the load from the continuous plies to the dropped region in the outer continuous plies through an offset in the z-(third) direction. He also noticed that the mismatch in the Poisson contraction in the continuous and dropped regions of the laminates with ply dropoffs can cause ply splits.

Samborsky et al. [54] studied the delamination of carbon and glass fiber prepreg laminates with ply drops under fatigue loading. They noted that the ply drop problem is more critical for carbon laminates due to more directional elastic moduli, a lower compression strength and thicker laminates. Samborsky and colleagues observed that delamination in ply drops occurs at relatively low applied strains under fatigue load, both for tension, compression and fatigue. The ply drop delamination would precede in-plane failure for all cycles and loading conditions for 0.3 mm or ply drops of a greater thickness with carbon 0o plies and for 0.6 mm in compression and between 0.6 mm and 1.2 mm in tension for glass plies.

In Ref. [37], the loss of strength in ply-drop areas in a wind turbine blade was studied numerically. The authors used modified Hashin-type damage criterion for the prediction of fatigue failure in a ply-drop submodel of a full blade model and estimated the failure indexes for different regions of the blade. They observed the interlaminar stress concentration near the resin pocket edges in the ply-drop area. A detailed overview of the methods of analysis of the tapered laminated composite structures is given in [50].

Generally, it can be concluded that the risks of delamination, fatigue and failure of the tapered areas of wind turbine blades can be minimized by choosing the optimal design, thickness and number of plies.

### 3.3. Structural Collapse and Buckling

The structural collapse of a wind turbine blade can occur if the blade is subject to extreme loads, exceeds its designed strength, and/or as a result of degradation and fatigue of the blade elements, which reduce its structural strength, thus making it more susceptible to extreme winds. As noted above, the structural integrity of a blade is ensured by the spar and shear webs, which are still quite strong under normal conditions.

The often-used approach to study the structural failure of wind turbine blades is the full scale **testing under flapwise load**, supported by **computational modelling**. Jensen et al. [55] carried out a full scale testing of a 34 m blade (with box girder) under flapwise loading measuring local deflections, and also computational modelling of the tests. The authors studied ovalization of the load carrying box girder under flapwise bending. They observed in their experiments and model that the debonding of the outer skin is the initial failure mechanism of the blade, which is followed by delamination buckling, leading in turn to the blade collapse. Jensen and colleagues also analyzed the Brazier effect [56] (non-linear deformation or “flattening” of the cross section) and observed that the crushing pressure caused by the Brazier effect has a strong effect on the web deflections.

Kühlmeier [57] tested a scaled-down prismatic blade section and observed that the interlaminar shear failure originated from local bending due to an initial geometric imperfection, which triggered a progressive collapse of the blade section.

Overgaard, Lund and Thomsen [58,59] studied the structural instability in the blades subject to flapwise loading as well. They developed a top-down approach, and a computational model, using the interlayer progressive failure finite element model. Overgaard and colleagues concluded that the sudden, progressive collapse of a blade is caused by the coupled phenomenon of delamination (interlaminar failure) and buckling, with compressive fiber failure in the delaminated flange material. Local delamination is initiated at the boundary of the adjoining structural elements and in the center of the compressive flange due to local buckling, leading to a sudden structural collapse and subsequent compressive fiber failure in the delaminated flange material. Specially, the effect of delamination on the compressive strength of thick laminates was investigated by Branner and Berring [60] They observed that large delaminations cause local buckling and failure (where the sublaminate buckles on one side of the delamination), while smaller delaminations closer to the surface can lead to stable delamination growth.

Yang et al. [61] carried out structural collapse testing of a 40 m blade under the flap-wise loading of blade, using the videometrics technique to estimate the blade deformation. They concluded that the Brazier effect was not the dominant failure mechanism for the tested blade. Rather, the shell debonding from the adhesive joints was the initial failure mechanism, which led to unstable debond propagation.

In Ref. [62], Chen and colleagues studied the failure of a 52.3 m blade under flapwise bending, and observed multiple failure mechanisms, in particular, laminate fracture, delamination, sandwich skin-core debonding, failure of sandwich core and the fracture of the shear web at the transition region. They identified the delamination of unidirectional (UD) laminates in the spar cap as the root cause of the failure of the blade. In another paper [63], Chen and colleagues employed finite element modelling, the lobal–local modeling approach and Progressive Failure Analysis (PFA) techniques to analyze the failure of a 52.3 m blade. They concluded that the accumulated delamination of the spar cap at the suction side and shear web failure at the transition region were the main reasons for the blade collapse. Chen and colleagues also stated that local buckling is an important factor which increases local out-of-plane deformation thus contributing to failure. The Brazier effect was not observed in the transition region. When the applied loads reached approximately 90% of the target test loads, the blade collapsed drastically, resulting in a significant failure in two distant regions, i.e., the root transition (RT) region and the near-tip region around the loading clamp (LC).

Al-Khudairi and colleagues [64] carried out a full scale fatigue testing of a wind turbine blade in the flapwise direction with an intact blade, and a blade with debonding cracks of different lengths (0.2 and 1 m) between the web and the spar cap. While the small cracks started to propagate even at 50% of the target bending moment (which the blade is expected to experience in its service life), and started only at 70% of the target bending moment, the large crack started propagate when the load exceeded the target bending moment, leading to failure.

Ullah et al. [65] investigated blade failure under large-deflection flap-wise bending in computational simulations and simulated the local skin buckling on compression side and the buckling-driven skin–spar debonding. They observed in their studies that the blade suction side subjected to high compressive stress can cause local skin buckling, which drives interfacial debonding between the skin and spar. At the same time, skin–spar debonding initiated in the stress concentration region at the adhesive interface prior to reaching the ultimate load propagated along the interface with an increasing load, leading to the redistribution of stress and local buckling. Skin–spar debonding from the adhesive interface is therefore the initial damage mechanism which can lead to the earlier failure of a blade. Ullah and colleagues employed linear buckling analysis, the submodelling approach and a cohesive zone model to simulate buckling-driven skin–spar debonding at the adhesive interface. In another study, Tang and Chen [66] investigated the failure of spar caps and the shear webs of blades under flapwise loading and also observed compressive crushing caused by the local buckling of shear webs. The critical failure mechanism of the box beams under edgewise loading was adhesive joint debonding triggered by local adhesive cracking and spar cap buckling.

More complex, realistic studies include both **flapwise bending and torsion effects**. Jensen and colleagues [31,32] noted that increasing the blade size, leading to increased edgewise loading due to the higher gravitational forces in larger blades can lead to the buckling of the trailing edge, and to fatigue failure in the root region. The torsional eigenfrequency becomes lower and the torsional mode may couple with some of the lower bending modes when the blade size is increased. This can cause flutter instability leading to blade collapse.

The combined bending and torsional deformation of blades was studied experimentally and numerically by Berring et al. [67]. Two 8 m blades were tested, and a 3D digital optical deformation measuring system (ARAMIS 2M and 4M) was applied to measure the displacement field on the blade surface.

Chen and colleagues [24] studied the collapse of a 47 m wind turbine blade under combined bending and torsion. They concluded that the root causes of the blade collapse were longitudinal compressive crushing failure, and the following spar cap delamination. These failure processes are driven by local buckling. They also concluded that the torsion load has no significant effect on the blade strength, but it does influence the post-collapse blade behavior. Zhang et al. [68] carried out failure tests of a 52.5 m wind turbine blade under combined loading (torsional and bending). Zhang and colleagues observed the blade damage mechanism, which starts by debonding and cracks in the trailing edge (due to bending load). The cracks formed, and the cracks further influenced the oblique bulge of the blade skin, formed due to the torsional moment. The combined mechanism of the buckling of the spar cap, the delamination of the panel and the debonding of the trailing edge leads to the catastrophic failure of the blade.

Thus, the identified root causes of buckling under flapwise loading include the Brazier effect, local buckling and delamination and debonding. Apparently, the role of each mechanism depends on the quality and strength of the corresponding elements (laminates, adhesive layers, interlaminar layers, interfaces). Given that the buckling process is strongly influenced by the properties of the interface if the composites [69,70], the conclusion can be drawn that the improvement of structural strength should include stronger interfaces and stronger/tougher adhesive layers in the blades.

### 3.4. Adhesive Joints in the Spar Area

As discussed in Section 2, the degradation, weakening and debonding of the adhesive layers (in the trailing or leading edges or on the spar/shell joint) is one of the main processes leading to wind turbine blade failure. Studies of the failure and strength of the adhesive joints of wind turbine blades are often based on research in related areas, such as the aerospace or automotive industry, where adhesive joints were used and investigated before they became some of the most critical elements of wind turbine blades [71,72].

A widely used approach to the analysis of adhesive joins in the wind turbine blade is **sub-component testing**. Here, the problem is reduced to the task of designing of a subcomponent (beam) which should reproduce the realistic loading conditions of the joint and testing the subcomponents under different loading conditions. Mandell et al. [51] studied the fatigue behavior of various sub-structural elements, representative of critical building blocks of wind turbine blade. They developed and tested five separate beam specimens, with three different configurations. They studied adhesive failure, and stress field in adhesives for different beam configurations and used both analytical and finite element simulations. To represent the spar area, Mandell and colleagues designed an I-beam with flanges and web. Sayer and colleagues [73] reviewed different testing procedures for adhesive joints and proposed asymmetric three-point beam tests (so-called Henkel-beam, combining 2- and 3-point bending), which provided the stress state in an adhesive bond, similar to the stress state in a wind turbine blade. In Ref. [74], Sayer and colleagues carried out fatigue tests of the adhesive joins using the Henkel-UpWind beam, representing the spar cap to the web joint. They observed tensile crack propagation transverse to the adhesive longitudinal axis, at 10% of the total lifetime, and the crack changed the direction resulting in disbonds between the adhesive–adherend interface at around 60% of the specimen life. Zarouchas et al. [75] studied prismatic composite I-beams subject to four-point bending tests and representing the adhesive between the spar caps and shear web, experimentally and numerically. They simulated the complex stress state and damage initiation in the adhesive bond line between the spar caps and shear web, using the progressive damage method developed by the authors and different failure criteria. In the simulations, they observed local failure in bonding paste I-beam close to the supports, and the loading points. Then, damage in the composite starts in the shear web skin due to matrix damage.

Typical joints in the wind blades can be relatively thick [76], reaching for instance 30 cm for 70 m blades [77]. The thickness, geometry and loading conditions influence the adhesive behavior. To take into account the thick adhesive effect, special specimens for bulk adhesives have been developed [77,78]. Samborsky and colleagues [76] studied the effect of adhesive properties, voids and geometry on thick (4 mm) adhesive joints specimens, using wedge block specimens (wedge/adhesive/web) and presented the results of 250 static and fatigue tests. The static strength of the specimen was shown to be similar for 45 and 90 degrees loading. Static tests were also insensitive to the test rate. Moreover, fatigue strength has a low sensitivity to angles. Zarouchas and van Hemelrijck [78] investigated the damage mechanisms of adhesives, using thick adhesive dog bone specimens under tensile and compressive static loading and applied acoustic emission and digital image correlation techniques to analyze the damage mechanisms. They observed three damage mechanisms in adhesives: development of micro-cracks in the adhesive, multiple matrix macro-cracks, and fiber breakage and pull-out. Zarouchas and Nijssen [77] studied the multi-axial loading effect on the adhesives in bonded joints using tubular specimens. They observed that the tension stress–strain response of an adhesive is not influenced by the biaxial ratio, while the compression and shear moduli were different for different biaxial loading cases. The thick adhesive testing allows a better understanding of the micro-mechanisms of adhesive degradation, and the material-related factors controlling the adhesive performance.

Finite element models of adhesive joints are often realized using the cohesive zone models. Sørensen et al. [79] studied the formation of a large-scale fracture process zone, including the crack tip and fiber bridging zone, using a cohesive zone model. Sørensen and colleagues analyzed the fracture of adhesive joints between two glass-fiber laminates by testing double cantilever beams. The authors observed an increased mixed mode fracture resistance with an increase in the crack length (R-curve behavior).

Hua et al. [80] studied carbon/epoxy spar–shell assembly under bending using the extended finite element method coupled with the cohesive traction separation law. The authors studied the effect of plasticity of adhesives and observed that the plastic behavior reduces the maximum peel and shear stress by 4…8%. They also studied the effect of voids (see Section 4). They observed that a larger shear modulus of the adhesive improves the bonding strength and reduces the fracture strength. Ji and Han [81] used the cohesive zone model approach to study the damage propagation at the skin–web adhesive joint in a blade under the flap-wise loading. Ji and Han observed that debonding forms in the edge of the adhesive layer prior to reaching the extreme load and propagates in the blade tip direction along the adhesive–web interface when the load increases. They also observed that shear stress is mainly responsible for debonding. Further, Ji and Han concluded that skin debonding from the adhesive joint is the initial mechanism of failure, which can lead to the earlier failure of the blade.

Raman and Drissi-Habti [82] carried out macro and meso scale simulations of failure in the adhesive material of a wind turbine under a quasi-static load (bending and torsion). In the meso-model, they simulated a single-lap-joint. In the macro-model, they simulated a 70 m blade using shell elements. They observed that the adherent layup orientations and properties have a strong influence on the strain distribution in the adhesive and joint failure. The multi-ply orientation made the failure less likely for the adhesive. A strong difference was observed for glass and carbon angle ply configurations. For carbon fiber laminates, a multi-ply orientation leads to less adhesive failure, while an angle ply leads to maximum failure value. For glass fibers, angle ply leads to less failure.

Jørgensen [83] studied the formation of transverse (tunneling, mode I) cracks in adhesive joints using the linear elastic fracture mechanics approach. In his experiments, he observed that that a higher post curing temperature led to the initiation of transverse cracks at a lower mechanical loading. The energy release rate of the tunneling crack could be slightly reduced by embedding a so-called buffer-layer (between the adhesive and substrate), and by reducing the thickness of the adhesive layer.

Adhesive joints in the spar area are critical elements of wind turbine blades, ensuring their high strength. Understanding the mechanisms of their failure and the factors which control the failure is necessary to enhance the blade lifetime and reliability. This understanding requires the development of sub-structure test specimens, which reflect the real loading conditions of the joints and also their real properties (finite thickness, non-linear material law, variations of interface strength, effect of plies, thickness, defects). In addition to the design of sub-component samples, reflecting the real service conditions, computational models, reflecting the cracking mechanisms, effect of thickness, plies are required for the analysis of the joints.

### 3.5. Adhesive Joints at the Trailing and Leading Edges

**Figure 6:**
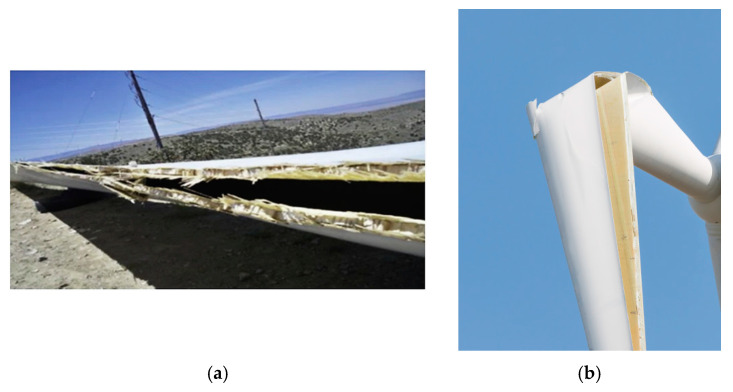
Blade failure: (**a**) Failure of edges in 24 m wind turbine blade. Reprinted from [27,84] with kind permission from Elsevier, and (**b**) Collapsed blade.

The trailing edge is another critically important structural part depending on the reliability of adhesive bond. Figure 6 shows the failed trailing edge of a 24 m wind-turbine blade.

Van Leeuwen et al. [85] tested a number of 3.4 m blades under static flapwise bending, flapwise fatigue and edgewise fatigue loading. Under the edgewise fatigue test, the blade failure started from the crack in the bonding at the trailing edge, and then the crack propagated in the laminate. It is of interest that the blade subject to the fatigue test failed at the tensile side, while the blade under a static load failed at the compressive side. While failure under flapwise fatigue load occurred at the tensile side, failure under edgewise fatigue was initiated in the adhesive of trailing edge, and led to crack growth in the laminate. The fatigue strength of blades could be well predicted by the classical Goodman relation for flapwise tests (not for edgewise tests). Raman and Drissi-Habti [82] simulated numerically the 70 m blade failure under static bending and torsion loading. They observed that the failure took place close to the root section and in the adhesive in the trailing edge. The trailing edge failure is caused by shear and torsion in the adhesive.

Mechanisms of trailing edge fractures were also studied by Eder, Branner, Haselbach and colleagues [86,87,88,89,90,91,92,93,94,95], using methods of fracture mechanics. Eder et al. [86] analyzed the debonding mechanism of the trailing edge joint by estimating the strain energy release rates (SERRs) in the trailing edges using the virtual crack closure technique. They concluded that mode III contributed the most to the strain energy release rate and the flapwise shear and torsion were the main contributors to the mode III fracture.

Haselbach and Branner [35] tested 1.5 MW SSP wind turbine blades and developed a computational model of damage in trailing edge. They demonstrated the interaction between trailing edge buckling and sandwich failure, which can lead to blade failure. The trailing edge is buckling sensitive, and even small imperfections can influence local buckling. The trailing edge deformation was observed to lead to core shear failure near the trailing edge. The buckling of the trailing edge caused damage in the sandwich composite panel on the pressure side, by bending and kinking. The trailing edge failure/debonding decreased the structural stability and reduced the blade stiffness. Cracks along the trailing edge were observed to occur on both sides of the adhesive bond but were also propagated through the middle of the bondline. In their further work, Haselbach and colleagues [87] studied the energy release rates at the tip of a transversely oriented crack in the trailing edge of the blade and demonstrated that a nonlinear wave can be formed in the trailing edge that can lead to the debonding of the trailing edge adhesive joint.

Lahuerta et al. [34,88] presented a blade sub-component test setup for studying trailing edge failure. They described three main stages of trailing edge instability (buckling), observed during the static test: pre-buckling, post-buckling (drop of the sub-component edgewise stiffness and nonlinear behavior until the final failure) and ultimate failure stage. They also listed various trailing edge strength and stability/knuckling resistance criteria, developed in [89]. Lahuerta and colleagues also studied the damage mechanisms under the fatigue test, and observed the failure mechanism, similar to that under the static test, with the formation of a buckling wave along the trailing edge.

Jørgensen and colleagues [90] simulated a tunneling crack in the trailing edge under fatigue, taking into account residual stresses in the adhesive, and predicted the crack growth rate. They observed that the energy release rate of a tunneling crack is relatively insensitive to the substrate thickness when the substrate stiffness is large, and also to specific layup near the adhesive.

Chen and colleagues [91] sought to provide an understanding of trailing edge failure in the subcomponent test, using the digital image correlation analysis of strain distribution and finite element modelling. They tested the trailing edge section cut from a 34 m full scale blade. Chen and colleagues observed that, in addition to the previously observed buckling-driven failure, two inside surfaces of the sandwich panels can come into contact during the load and this has a strong influence on the failure of the trailing edge sections.

Adhesive joints of the leading edges of wind turbine blades are not among the most often observed damage sites. Still, it is an important structural region which can potentially degrade. The degradation of the adhesive joint region has been studied in several works [93,96]. Droubi et al. [96] used cohesive zone modelling methods to investigate the elastic indentation contact of adhesively bonded leading-edge composite joints in wind turbine blades. They compared different adhesive joint designs, including single lap, ideal, stepped, double scarf designs. They observed that mode-I opening is the dominant cause of fracture in joints, with fracture initiation locations in the areas of tensile stress. In lap joints, they observed the stresses above the strength values (shear strength, ultimate tensile stress, compressive strength) of glass fiber reinforced epoxy composites, suggesting that there would be the possibility of composite failure before adhesive bond failure.

### 3.6. Root Region

Failure of the root end of the wind turbine blade is another critical failure mechanism. As a result of root end failure, the blade can be pulled from its hub during the service time. Lee et al. [97] reported that blade failures observed in the field at Eclipse and Ocotillo wind farm in 2013 were caused by pulling out the blades from the wind turbines due to delamination at the root. Lee and colleagues also observed this mechanism in the testing of 30 m blades and in their simulations. On the basis of the simulations, Lee and colleagues made the conclusion that the load distribution at the root of a slender and large wind turbine blade is very different from the distribution, which was obtained from the model of root as a hollow circular cylinder subject to bending. They observed delamination failure at the end of the blade root, due to local load increase, causing a partial separation of the T-bolt joints leading to the delamination at the end of the root.

In their studies of failure reasons of blades, Marin et al. [13] observed cracks in root to airfoil transition area and identified the root causes of damage as an abrupt change of thickness, local geometry of stress concentrator in the transition area and manufacturing defect (lack of resin in some regions, debondings).

Hosseini-Toudeshky et al. [98] studied progressive debonding of the blade root adhesive joint of a 660 kW wind turbine under static and cyclic loading, using a cylindrical model with metallic, composite and adhesive parts and cohesive zone modelling. They observed that when the blade is overloaded ~10 times of its normal fatigue loads, considerable damage growth will occur in the adhesive bonding of the root joint after 1 million cycles of fatigue loadings (~1 month).

Again, one can see that the root cause of the failure of the root region of wind turbine blades is related to delamination and adhesive debonding, i.e., with interface and thin layer effects.

Summarizing the above investigations, one can make the following conclusions:

- Apart from leading edge erosion (which is a multiphysical, mainly microscale process), the failure mechanisms of wind turbine blades are caused by interface debonding or thin layer (interlaminar layer, adhesive layer) degradation, which can trigger the buckling of composites under compressive loading. It is known that the buckling of composites is strongly influenced by interface strength, shear modulus of matrix near the interface and the angle between the compressive load and the interface [99]. Given the strong influence of interface properties on the buckling, one can state that the role of strong, tough interfaces in blade composites and structures cannot be overestimated.
- Leading edge erosion is the most prominent example of microscale processes, which in fact triggers the drastic reduction in the performance of wind turbine blades, and can lead to failure of the full blade. These environmental effects (temperature variations, moisture effects, etc.) as well as small, random load variations have an apparently stronger effect on other mechanical failure mechanisms.
- The influence of even small deviations in the geometry on the failure likelihood is quite strong. In the next section, we consider the available data on the influence of the manufacturing defects on the failure of wind turbine blades.

One should note that the damage mechanisms of wind turbine blades are strongly influenced by environmental effects such as humidity, temperature variations and ultraviolet (UV) radiation. In this review, these effects were not discussed, due to very few sources in the literature and very few investigations devoted to the environmental degradation of wind turbine blades.

## 4. Manufacturing Defects and their Influence on Blade Failure Mechanisms

The failure of wind turbine blades is influenced by design peculiarities (for instance, tapped and transitional region), high mechanical and environmental loads and material limits. However, there is more and more evidence that manufacturing defects are among the main factors limiting the potential strength and reliability of the blades [17]. This can be easily explained. The target price for manufacturing wind turbine blades is 5 USD/lb (7 GBP/kg), while for aircraft and other composite structures the target prices are of the order of 100–1000 USD/lb (the data are from 2013 and have changed since then) [17]. The lower price is also reflected in the manufacturing and product quality.

### 4.1. Common Manufacturing Defects of Wind Turbine Blades

**Figure 7:**
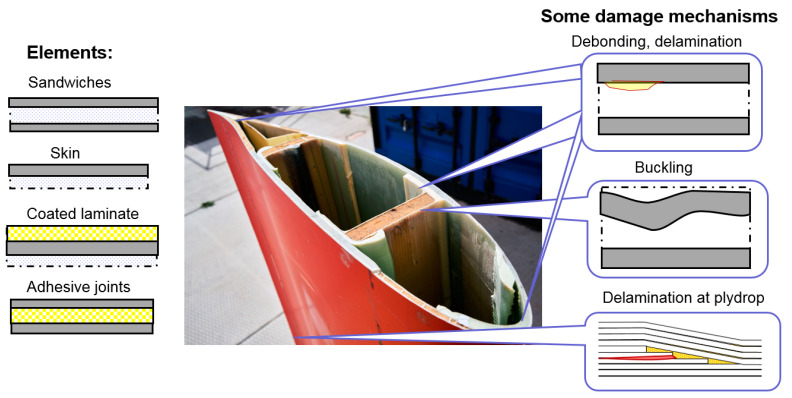
Elements of the blade structure (**left**), blade cross section (**middle**) and some common damage mechanisms (**right**). On the blade cross section, white adhesive layers and brown/white sandwiches can be seen. The photo from Bigstock© collection is reproduced according to Bigstock standard content usage agreement.

Wind turbine blades are structures built from fiber-reinforced composites, including various elements such as single skins, sandwich structures and adhesive layers [38]. Figure 7 (middle) shows a cross section of a wind turbine blade, with white adhesive layers and brown/white sandwiches in shear web and shell.

The complexity of the structure leads to a situation where the smallest deviations from the designed geometry of blades can drastically influence the blade life. In particular, internal defects in the elements of the blade can reduce the lifetime of wind turbine blades and trigger various failure mechanisms. Figure 7 shows the main elements of a blade (sandwiches, adhesive joins, skin) and some typical structural blade failure mechanisms (debonding, buckling, delamination, ply-drop delamination).

Cairns and colleagues [100] categorized flaws as “design flaws” (e.g., ply-drops) and manufacturing flaws. The following defects are considered as manufacturing flaws [62]: porosity, debonding, delaminations, improper fiber/matrix distribution, fiber misalignment, improper fiber/resin ratio, bonding defects, foreign inclusions, incompletely cured matrix and matrix cracking.

According to [17], the following internal defects are observed in blades: thickness variability and voids in adhesive bonds, ply wrinkling and waviness, misplaced laminates, fiber misorientation and misalignment and heterogeneities (resin rich regions and dry spots). The manufacturing defects can be formed due to imperfect curing conditions, air trapping in polymers, misplacement of fibers or layers, not enough adhesives and so on. Voids and bubbles can be formed in the polymer matrix, adhesive layers and coatings due to mechanical air entrapment during resin flow and from gas created due to chemical reactions during the cure and nucleation of dissolved gases in the resin [101]. Under loading, voids or bubbles act as notches, creating a local stress concentration. For instance, a local stress near a round void under a tensile load can be 3 times higher than remote stress. Debonded regions form in the coatings, adhesives or in laminates due to bad adhesion and serve as initiation points for crack growth. Misaligned fibers as well as weak interfaces drastically reduce the compressive strength of composites, triggering the buckling of composites (for instance, spar composites under compression load).

**Figure 8:**
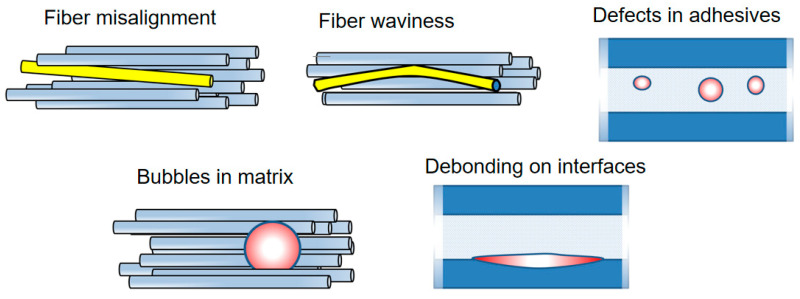
Schema of the typical manufacturing defects on wind turbine blade composites: fiber misalignment and waviness, voids in adhesives and in matrix, debonding on interfaces.

Figure 8 shows schematically the common defects observed in wind turbine blade composites: fiber misalignment and waviness, voids in adhesives and in matrix and debonding on interfaces.

### 4.2. Blade Failure Mechanisms and Role of Manufacturing Defects

In a number of works, the direct connection between the availability of manufacturing defects and the degradation of wind turbine blades has been observed.

Generally, manufacturing defects are formed in random locations and their formation is a random event. Riddle and colleagues [102] proposed probabilistic models to assess the reliability of a wind blade with known defects. Considering defects as random variables and design parameters in a parametric probabilistic analysis, the authors demonstrated that safety factors may be reduced, thanks also to a reduced uncertainty of the blade failure. Toft and colleagues [103] presented two stochastic models for the distribution of delaminations in wind turbine blades (completely random and clustered, defined by two coupled Poission processes) in main spars. They assessed the reliability of a blade with defects, using first and last ply failure conditions and found that the probability of failure increases drastically when defects are included, especially for clustered defects. However, the rather complex blade design different defects and different acting damage mechanisms requires more detailed attention to the specific defects and their locations. In the following, typical defects in various critical regions of wind turbine blade and their effect on the damage mechanisms are reviewed.

**Figure 9:**
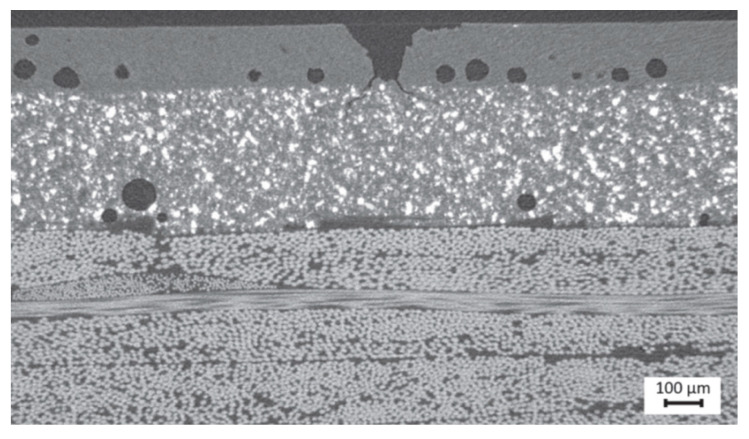
Voids in leading edge protection layers: Sites of damage initiation. Reprinted from [15] with kind permission from Wiley.

**Leading edge erosion.** The microscale mechanisms and factors controlling the leading-edge erosion have been studied in [15,16,48,49]. Wind turbine blades from old wind turbines from Vindeby wind farm, established in 1991 and decommissioned in 2015–2017, were cut and investigated using X-ray tomography [15,16]. The interesting observation was that the erosion damage starts in fact not from the contact surface (where a rain droplet hits the blade and the highest stresses are expected), but from voids and debonded particles inside the coating. Figure 9 shows the voids in the leading edge coatings and the start of the damage from the voids. Further, computational models of coatings have been developed, which include the real observed defects inside the coatings [15,16]. In the computational simulations, it was again observed that the erosion microcracks start from voids or debonded particles in the coatings, and not from the high stress regions of the contact area on the coating surface (as one could expect). In Refs. [48,49], the authors ran simulations of rain droplet impact on intact and voided coatings and observed that even small voids drastically increase local stresses, and reduce the lifetime of coatings.

**Spar caps, shell, parts** [20]. There can be various defects in composites, which reduce their strength including wrinkles, misalignments, waviness, delaminations, voids in matrix and debonded interfaces.

*Wrinkles* can be observed in thick composite parts, e.g., in sandwich panels. They can drastically reduce the compressive strength of composites, with up to 55% strength reduction for sandwiches [104,105]. Leong et al. [104] studied numerically the deformation and failure of laminate/balsa core sandwich specimens with face sheet wrinkle defects, under in-plane compression loading. They observed different mechanisms of failure, namely, debonding between the face sheet and the core, in the vicinity of the wrinkle; failure of the face sheet with the wrinkle defect by layer-wise delamination through the laminate thickness and total specimen failure. Bender et al. [106] presented a numerical model of wind turbine blade sub-structure with a tapered beam and a wrinkle under tensile loading and demonstrated that the parameters of wrinkle geometry (angle, depth, location) have a strong influence on the failure initiation and delamination conditions.

Other common defects in laminates are *delamination defects.* Li et al. [107] studied the effect of delaminations on the strength of UD laminates of spar cap, using finite element models of laminate with different delaminations (delamination close to the midsurface or close to the surface areas). They observed that the larger the delamination area is, and the closer the delamination is to the surface, the more likely it is to cause buckling failure. The failure mechanisms of laminates with delamination defects were local buckling under the load, and mixed mode of local buckling and global buckling under a heavy load.

*Ply waviness* in the laminate can reduce the compressive strength due to the misalignment effect, increasing the likelihood of buckling [69,70], and also due to more matrix-dominated damage mechanisms [108]. Fiber misalignment has a strong effect on the compressive strength of composites: by increasing the standard deviation of random distribution of fiber misalignment angle from 1 to 1.4 degree, the damage level in the fibers can increase by 3 times [69,70].

Avery et al. [108] investigated the effect of fiber waviness on compressive strength, testing glass and carbon laminates with plydrops, joints and inclusions, and observed the strain to failure reduction from 1% (straight carbon fibers) to 0.6…0.8% (wavy fibers). They observed that epoxy resins are less sensitive to the waviness than vinyl ester resins with carbon fibers. Single ply drops in prepreg laminates lead to static ultimate compressive strains 0.70…0.75%.

Nelson and colleagues [109,110] studied the effect of in-plane (on the surface) and out-of-plan (through the thickness) waviness and porosity of composites, using testing of coupons with flaws and damage modelling (continuum and discrete damage modelling). They observed that the strength degradation in laminates with waves correlates well with the average of the maximum fiber misalignment as measured through the thickness.

Further, the availability of *voids* or defect-caused softening of matrix leads to a reduction in the compressive strength of composites [69,101]. Nelson and colleagues [109,110] studied the effect of porosity of composites as well and observed that voids lead to a reduction in the bulk modulus of the polymer, thus reducing the compression strength of the composite. A total of 10% porosity led to a 20% reduction in the failure stress. The authors concluded that the acceptable porosity limit was 2%.

**Adhesives bonds.** Katnam et al. [111,112] observed that “micro-voids are frequently seen in adhesive bonds cured at elevated temperatures” and showed that the tensile strength depends on the micro-voids. Voids are formed in epoxy resin during curing [113]. In [114], it was demonstrated in numerical experiments that voids in adhesives create local stress concentration, increasing local stresses ~2 times…3 times higher than in the material around.

Hua et al. [80] simulated the stress distribution in an adhesive layer with elliptic cylinder, representing entrapping air bubbles, and observed a significant reduction in the strength and more than two-fold increase in the magnitude of interlaminar stresses in the adhesive layer. They also studied the fracture behavior of adhesive joints, with flat adhesive, filleted adhesive with and without through-thickness imperfection. Hua and colleagues observed in simulations that cracks initiated in the upper portion of the adhesive layer and propagate following a path immediately adjacent to the interface. The strength of the adhesive joint was reduced by 2.4% and 4.8%, respectively.

Samborsky et al. [76] presented the results of static and fatigue tests of thick adhesive joints specimens, representative for blade joints. They studied and simulated adhesives with voids and flaws and observed that that crack initiated at the flaw areas in the adhesives, shifting to interlaminar in the adherend as the crack propagated.

Sørensen and colleagues [21] simulated crack growth in adhesive joints with air bubbles. They considered two plates, bonded in the overlapping region, with the bond containing a periodic arrangement of elliptic or circular voids, and simulated quasi-static crack propagation, using cohesive law model. They observed, as expected, that the strength of bond was reduced when the crack approached a voids.

Sayer and colleagues [74] carried out fatigue tests on Henkel-UpWind beams (representing spar cap to web bondlines) with artificial flaws (cylindrical holes in the middle of the bond line in the highest loaded area, a notch on the hole surface, incipient interface disbond. Surprisingly, they observed a negligible impact of the flaws on the fatigue life.

**Trailing edge:** Trailing edge buckling is very sensitive to local defects [35]. Local buckles and flaws (entrapped air-bubbles or badly bonded areas) in trailing edge bond line can trigger the cracking under loading [92]. Chen et al. [115] carried out fatigue testing of a 14.3 m blade with four types of artificial defects, introduced into the blades (laminate wrinkles in spar caps, core materials/skin laminates debond in sandwich panels, adhesive joint debonds in trailing edge, and adhesive joint debond between the shear web and the spar cap), subject to flapwise and edgewise loading in sequence. As expected, delamination damages started from embedded wrinkles in the spar cap laminate and grew steadily until the blade failure by one delamination region. Moreover, the debond of adhesive joints in the trailing edge and between the shear web and the spar cap developed, while the debond between skin laminates and core materials in sandwich panels did not cause damage growth.

Eder and Bitche [92] studied fracture of adhesive bond in trailing edge of blades, paying special attention to the influence of imperfections and local buckling in the adhesive bonding line. They simulated the crack growth in the trailing edge in a blade submodel for different crack lengths. Eder and Bitche concluded that local buckling (of both the suction and the pressure side shell) induces Mode-I crack opening in the interface and leads to debonding with unstable crack propagation.

From this (non-exhaustive) overview, one can conclude that the manufacturing defects play critically important roles for the degradation and failure mechanisms of blades. The defects, voids, debondings and misalignments serve as sites of damage initiation, or weaken the strength of blade elements, reducing the critical strength or lifetime.

## 5. Structural and Design Solutions for Preventing or Resisting the Damage Mechanisms of Blades

In this section, some technical solutions which seek to prevent various damage mechanisms of wind turbine blades, are discussed.

### Leading Edge Erosion

Available solutions for preventing or counteracting the leading-edge erosion of wind turbine blades can been grouped as prevention and avoidance (e.g., by reducing the tip speed during heavy precipitation events [116]), repair (placing thick protective shields or tapes on the eroded area), protection (e.g., by developing highly protective advanced coatings) [39]. In Ref. [116], the authors proposed a so-called “erosion safe mode control” strategy of the mitigation of leading-edge erosion based on the reduction in the tip speed during heavy rain conditions. Since extreme precipitation events occur quite rarely, they suggested that the tip speed is reduced during such extreme periods.

Solutions for the development of new, highly protective coatings for wind turbine blades include multi-layered, highly damping, particle, nanoparticle or fiber-reinforced polymer coatings, ensuring the scattering of rain impact energy and additional toughening and damping mechanisms, but also special metallic coatings [117]. Variations of coating thickness, internal structure (e.g., interpenetrating polymer networks), variations of viscoelastic properties are among other parameters which allow the erosion resistance of coatings to be increased [39]. Thicker, highly damping, multilayered, tougher coatings allow increased erosion protection [117].

Johansen et al. [118] tested anti-erosion coatings with graphene and graphene-silica hybrid reinforcements, using single point impact fatigue test (SPIFT), and observed that the nanoengineered coatings ensure up to 13 times higher lifetime than pure polyurethane coatings. Jespersen and colleagues [119] demonstrated in computational simulations that Kevlar pulp reinforced polymer coatings have the potential to ensure better anti-erosion performance than the homogeneous polymer coatings.

**Buckling resistance and spar cap.** An approach to improve the buckling resistance of a spar includes using **stronger, more resistant flanges**, e.g., sandwich, hybrid or carbon plies.

Berggreen and colleagues [120] studied the possibility of using sandwich construction in the flanges of spar of extra-large blades, using global and submodelling finite element models of spar. They demonstrated that the sandwich structure (instead of single skin) in the spar allows the blade weight to be reduced and the buckling strength to be increased. The method for increasing the buckling resistance and weight reduction based on using sandwich structures [121] can however lead to the decrease in the flapwise stiffness and natural frequency and increasing costs.

Cox and Echtermeyer [121] sought to improve the buckling resistance of blades by introducing carbon fiber plies in addition to glass fiber plies. They carried out an FE buckling study of a 10 MW reference blade, with different composite layups, including carbon and glass plies, and investigated the relationships between plies orientation and buckling behavior. They demonstrated that the stability of the ply orientation influences the buckling failure location and the failure mechanism. While the Brazier effect (and thus the lower failure strain) was observed for the unidirectional 0° ply layup, it was partially suppressed for higher stability ply orientations (±60°…90°), leading to a higher buckling load. Cox and Echtermeyer concluded that “implementing 0° carbon with higher angle glass fiber plies is a cheap and simple technique to improve the stability of the blade”. Rosemeier and Bätge [122] investigated a carbon spar design for an 80 m blade, comparing continuous spar cap and split spar cap concepts, and lay-ups with hybrid spar caps or sandwich. They carried out parametric studies, evaluating the buckling resistance and tip deflection, and observed that split spar caps led to the lowest blade mass and increased the edge-wise stiffness.

Another approach is based on introducing **additional reinforcing elements**, which prevent the buckling possibility of caps. To reduce cap failure due to interlaminar failure, Jensen and colleague [31,32] proposed the use of so-called transverse cap stiffeners. An internal wire reinforcement should allow the out-of-plane deflection of the panels to be prevented, reducing the peeling stresses and impeding the Brazier effect [123]. In the concept of 100 m blade, presented by Griffith and Ashwill [124], a third shear web was proposed, beyond midspan, located within the aft panel region, which ensured an increased buckling resistance. The design also incorporated trailing edge and leading edge reinforcements, with the goal to improve the edge-wise stiffness. Ghasemnejad et al. [125] suggested to use z-pinning with natural flax yarn on composite beams, to increase delamination resistance and postpone failure process.

In several works, **methods of topology optimization** were used to improve the blade design. Lund et al. [126] applied the Discrete Material Optimization approach to maximize the buckling load factor of laminated hybrid composite shell structures. They observed that the unidirectional CFRP material should be placed at the top of the spar, and distributed through the thickness where the webs join the spar cap.

Blasques and Stolpe [127] presented a beam finite element formulation for the analysis of anisotropic beams with arbitrary cross section geometries and formulated a minimum compliance multi-material topology optimization problem for this task. They used this approach to optimize the load carrying structure of the blade and determined the optimal material distribution and laminate properties in a profile cross section. In Ref. [128], Buckney and colleagues also applied topology optimization techniques to 45 m blades. They suggested a layout that varies along the blade length. Then solution includes two spar caps, initially at the point of maximum thickness and becoming offset to the tip.

Buckney et al. [129] investigated various structural layouts of blades using topology optimization techniques. They proposed structural shape factors to evaluate structural concepts with view of maximal stiffness and minimal stress for asymmetric bending. The alternative layout suggested includes trailing edge reinforcement and the offset spar cap topology.

Sjølund, Peters and Lund [130,131] used structural gradient-based sizing optimization to minimize the mass of a 73.5 m blade, with ply-group thicknesses as design variable and buckling as criteria. The approach allowed the shell thickness to be optimized and the mass of the blade to be reduced.

One should also mention here the concept of **bend-twist coupling of wind turbine blades** [132,133]. The incorporation of bend-twist coupling into blades realizes the so-called passive load control, i.e., the modification of blade design which should ensure lower loads. This design can be realized as geometric-based coupling, which uses a sweep along the blade to create a moment that induces a twist, or as a material-based coupling, which aligns the primary load-carrying spanwise fibers in an off-axis manner [132]. Ashwill [132] demonstrated a blade, with incorporated off-axis carbon in the blade skin, to ensure bend-twist coupling, and observed enhanced strength of the blade. In Ref. [134], the feasibility of using braided composite preforms to manufacture a blade were studied, and it was demonstrated that a braided composite blade can be manufactured with the required coupling properties.

**Trailing edge:** The idea to use the additional reinforcing elements is also applicable to the trailing edge. Jensen and colleagues [31,32] proposed the so-called Bladena enhancer (deformation limiting wire solution), linking the sides of shell near the trailing edge, to reduce deformation and counteract fatigue failure in adhesive joint of trailing edge. The deformation was shown to be reduced by 30…40%. Chen and colleagues [135,136] carried out computational and experimental analysis of trailing edge failure mechanisms. They concluded that the contact constraint between internal surfaces of sandwich panels increases the buckling resistance of trailing edge and proposed to connect the surfaces using either core materials or adhesives, just at the location of expected contact, to improve buckling resistance.

With view on improving residual strength of trailing edge of blades, Lee and Park [23] proposed the use of the [±45] inner layer, which should have contact with the adhesive instead of the [0] inner layer.

Raman et al. [27] and Raman and Drissi-Habti [84] optimized composite layup, ply thickness and trailing edge structure, to prevent damage in root and trailing edge area under flapwise bending torsion load. For trailing edge, they suggested the use of the so-called “carbon composite cord stitching” (stitching fibers, as composite cords introduced in adhesive bonding zone which should tie together the structure). Moreover, Al-Khudairi and Ghasemnejad [137] studied the effect of stitching on failure of joints in blades (T-and box-beams with adhesive and stitches), and observed that stitching clearly improves the fracture resistance of the joints.

**Transitional regions.** The delamination risk from tapered laminates can be reduced by using gradual, staggered plydrop [138] placement of dropped plies between continuous plies, introducing an adhesive layer at the ply drop region [138,139]. Cairns et al. [52] noticed however that it is hardly realistic to suppress the delamination in service conditions completely.

Cairns and colleagues [52] carried out a comprehensive experimental study of delamination of tapered laminates and also tested delamination prevention techniques, ‘Z-Spiking’ (i.e., “removing the binder from the fabric and driving the fiber tows into the lower layers” [52]) and feathering (when “alternating tows were pulled some distance past the adjacent tows to provide a less defined ply drop front”). They observed that both ‘Z-Spiking’ and feathering reduced the delamination growth rate.

Khan and colleagues [138] proposed the use of ply edge chamfering technique (“putting a chamfer on the ply edges by abrading them with fine grit-sized abrasive media”). Gouldstone et al. [140] proposed reinforcing ply drop interfaces with vertically aligned carbon nanotube forests, and demonstrated that the nano-reinforcement of the interlaminar region prevented failure along the ply interfaces, and mitigates weakness at ply interfaces.

Moreover, with view on root region, Jensen and colleague [31,32] proposed the modification of the root area and transition zone of blades with a load distribution floor. This allows fatigue degradation caused by increased edgewise loading and higher gravitational forces in larger blades to be prevented.

**Figure 10:**
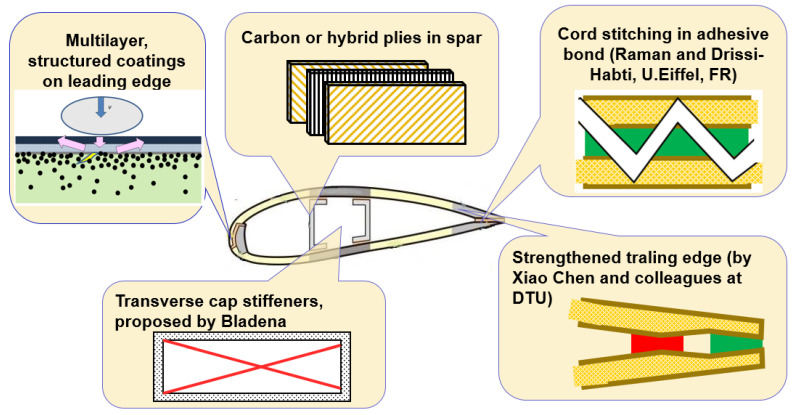
Schema: Some technical solutions for preventing or mitigation of different damage mechanisms of wind turbine blades, discussed in Section 5: (**upper left**) multilayered, architected coatings to mitigate leading edge erosion [39,117] (the figure shows the blade coated with multilayer leading edge protection, reinforced by black particles, with rose arrows showing the stress wave direction after rain droplet impact) (**upped in the middle)** carbon or hybrid plies in spar laminates, increasing the strength [121], (**lower left**) transverse stiffeners proposed by Bladena to prevent buckling [31,32], (**lower right and upper right**) strengthened trailing edge by Chen and colleagues [136] and cord stitching in adhesive layers, preventing the layer degradation [84].

Figure 10 shows schematically some technical solutions for the prevention or mitigation of different damage mechanisms of wind turbine blades, discussed in this section.

Summarizing, one can state that different reinforcing elements in wind turbine blades can allow the common damage mechanisms to be mitigated and reduced. They can be realized on structural level (such as internal wires, split spar caps, transverse cap stiffeners or third shear web), or at material level (vertically aligned carbon nanotube forests, reinforcing the interlaminar region). Moreover, the topological optimization allows the blade structure to be improved, taking into account several key criteria (such as weight and buckling resistance).

## 6. Conclusions

Failure mechanisms of wind turbine blades and their root causes have been reviewed in this paper. The methods of analysis of failure mechanisms of blades include a post-mortem analysis of failed blades (visual, microscopy, testing), full scale testing of blades in laboratories, with video-observation, structural health monitoring, analysis of data bases and collections of incident reports, direct monitoring of blade deformation and degradation during service using attached or embedded sensors and computational modelling of blade deformation and damage, and combinations of all these methods. With view on specific damage mechanisms in specific regions, testing of subcomponent (e.g., beam), reproducing parts or elements of blades (e.g., joints or sandwiches), or specific physical process testing (e.g., rain erosion testing) are employed.

The often-observed damage mechanisms include leading edge erosion, adhesive joint degradation, trailing edge failure, buckling and blade collapse phenomena. Critical areas of wind turbine blades include the outstanding and high velocity region (blade tip, leading edge), transitional and tapered areas (plydrops, root region) and interface regions (adhesive joins in spar/shell, trailing edge).

Methods of investigation of failure mechanisms of wind turbine blades include the full scale testing of blades in laboratories, a post-mortem analysis of failed blades, analysis of incident reports and computational modelling. The most endangered regions of the blades include the outstanding and high velocity region (blade tip, leading edge), transitional and tapered areas (ply drops, root), interface and bonded regions (interlaminar layers, adhesive joints in trailing edge, cap/shell).

**Critical role of interfaces and bonding layers:** Apart from leading edge erosion (which is a multiphysical, mainly microscale process), the failure mechanisms of wind turbine blades are often triggered by interface debonding, delamination and thin layer (interply, adhesive layer) degradation, which interact with composite buckling processes. Given that the buckling of composites is strongly influenced by interface effects, the role of strong, tough interfaces in blade composites and structures cannot be overestimated. The critical roles of interfaces mean that the development of new technologies of bonding, surface preparation and bond quality control is an important element for the blade life extension.

**Manufacturing defects and quality of blade elements:** The role of manufacturing defects in the failure mechanisms of wind turbine blades was discussed. The flaws can be classified as “design flaws” (e.g., plydrops) and manufacturing flaws. The manufacturing flaws include porosity, debonding, delaminations, fiber misalignment and waviness, ply wrinkling, bonding defects, foreign inclusions, incompletely cured matrix, thickness variability and voids in adhesive bonds, heterogeneities (resin rich regions and dry spots). The manufacturing defects can be formed due to imperfect curing conditions, air trapping in polymers, misplacement of fibers or layers, not enough adhesives and so on. Under loading, these defects act as notches or microcracks, creating local stress concentrations and serve as initiation points for growing cracks. The critical role of manufacturing defects means that several critical directions for the development of high-quality blades, namely, the automatization of manufacturing and quality control of blades and improved manufacturing technologies are especially important for the future. A number of technical solutions exist to reduce different damage mechanisms, which can be realized on a structural level (deformation limiting wire solutions and stiffeners, e.g., by Bladena), split spar caps, transverse cap stiffeners or third shear web), or at the material level (vertically aligned carbon nanotube forests, reinforcing the interlaminar region).
